# PI3K inhibitors in inflammation, autoimmunity and cancer

**DOI:** 10.1016/j.coph.2015.05.017

**Published:** 2015-06-18

**Authors:** Anne-Katrien Stark, Srividya Sriskantharajah, Edith M Hessel, Klaus Okkenhaug

**Affiliations:** 1 Laboratory of Lymphocyte Signalling and Development, Babraham Institute, Cambridge CD22 3AT, UK; 2 Refractory Respiratory Inflammation Discovery Performance Unit, Respiratory Therapy Area, GlaxoSmithKline, Stevenage, Hertfordshire, UK

## Abstract

The healthy immune system protects against infection and malignant transformation without causing significant damage to host tissues. Immune dysregulation results in diverse pathologies including autoimmune disease, chronic inflammatory disorders, allergies as well as immune deficiencies and cancer. Phosphoinositide 3-kinase (PI3K) signalling has been shown to be a key pathway in the regulation of the immune response and continues to be the focus of intense research. In recent years we have gained detailed understanding of PI3K signalling, and saw the development of potent and highly selective small molecule inhibitors, of which several are currently in clinical trials for the treatment of immune-related disorders and cancer. The role of PI3K signalling in the immune response has been the subject of detailed reviews; here we focus on relevant recent progress in pre-clinical and clinical development of PI3K inhibitors.

## PI3K signalling

**Figure 1:**
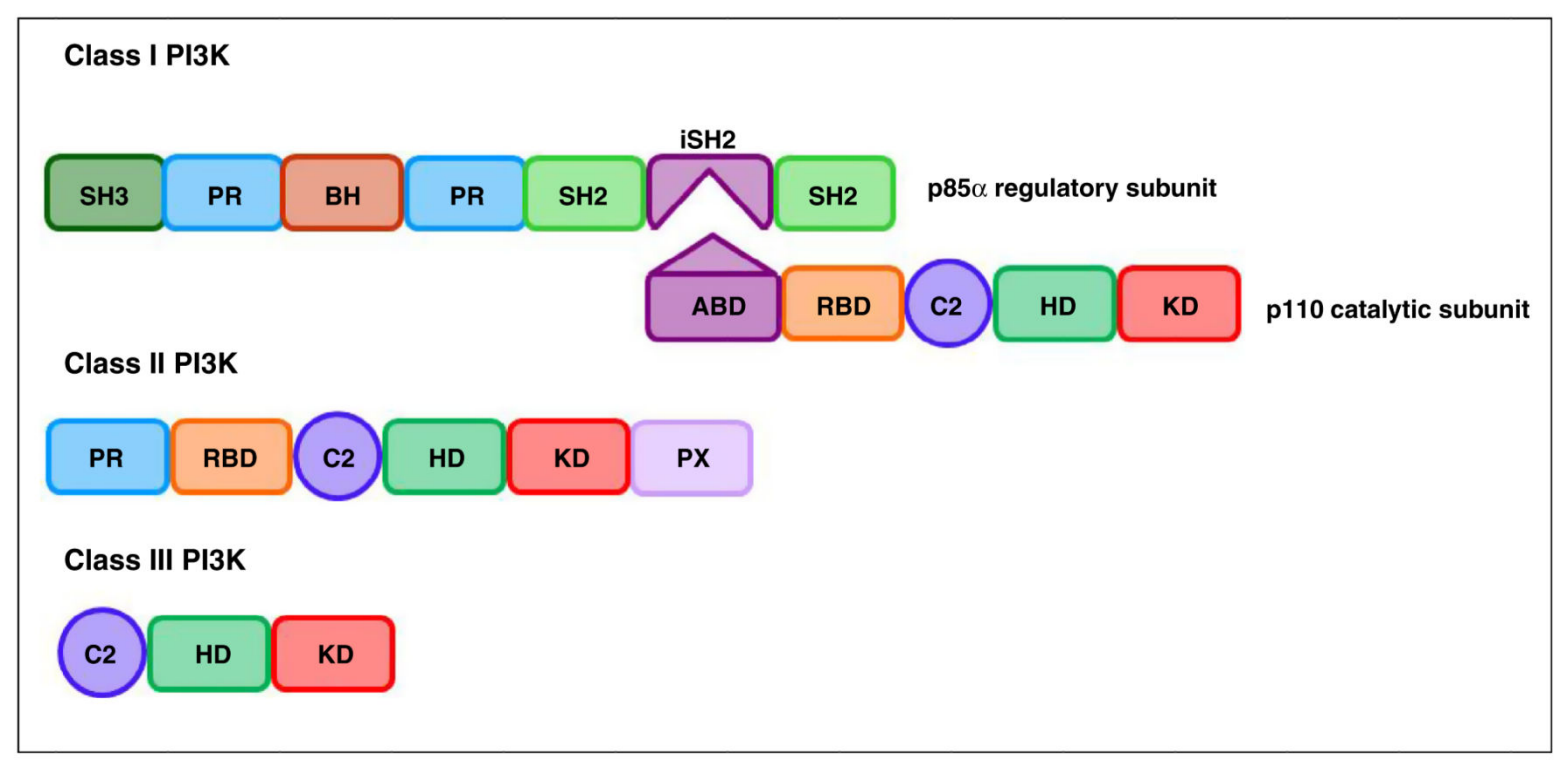
Schematic representation of class I-III PI3K structures ABD: adaptor binding domain; RBD: RAS binding domain; C2: C2 domain; HD: helical domain; KD: kinase domain; PR: proline rich domain; PX: phox homology domain; BH: breakpoint cluster region homology domain (Rho-Gap-like domain); iSH2: inter-SH2 domain (p110 binding domain). Complexes between p110α, p110β, p110δ and p110γ and their respective regulatory subunits are often referred to as PI3Kα, PI3Kβ, PI3Kδ and PI3Kγ.

The PI3Ks are a family of lipid kinases that phosphorylate the 3rd hydroxyl on phosphoinositides in cell membranes. Structurally, these enzymes share a common PI3K core motif, consisting of a C2 domain, a helical domain and a catalytic (kinase) domain. PI3Ks are classified into three families based on structure and substrate specificity, with the class I PI3K being further subdivided into class IA and class IB, summarised in Table 1 and Figure 1 [1–3].

## Class I PI3K

Gene targeted mouse models exist for all the class I PI3K catalytic and regulatory subunits and, together with the availability of isoform specific inhibitors, have greatly enhanced our understanding of PI3K signalling. Class I PI3K function as heterodimers consisting of a regulatory subunit associated with a catalytic subunit and phosphorylate PI(4,5)P_2_ to form PI(3,4,5)P_3_ which recruits pleckstrin homology(PH)-domain containing effector proteins such as AKT (PKB) to the cell membrane [2]. Under most circumstances class IA enzymes are activated through receptor tyrosine kinases (RTK) and other tyrosine kinase coupled receptors, while the class IB isoform p110γ is activated through G-protein coupled receptors (GPCRs). However, this distinction is becoming increasingly unclear: p110β can be activated by GPCRs [1,2], and one study found that p110γ function downstream of RTK, TLR and type-I cytokine receptors [4].

Class I PI3Ks play an important role in immune regulation, and the four isoforms differ in terms of tissue distribution and function: PI3Kα is ubiquitously expressed and essential for angiogenesis and insulin signalling [5]. PI3Kα can also compensate for the loss of PI3Kδ during early B cell development [6]. Like PI3Kα, PI3Kβ is ubiquitously expressed, but plays a non-redundant role in Fcγ receptor-dependent phagocytosis and ROS production in macrophages and neutrophils [7,8]. PI3Kδ and PI3Kγ expression is mainly restricted to leukocytes, and their expression levels and function vary based on cell type and activation conditions. PI3Kδ function is critical for mature B cell development as well as effector T cell and regulatory T cell (Treg) differentiation and function [6,9–11]. PI3Kδ and PI3Kγ can act synergistically to modulate myeloid effector function: sequential PI3Kγ and PI3Kδ activation is required for effective ROS production in human, but not mouse neutrophils [12], and aberrant migration in aged neutrophils could be partially corrected by PI3Kδ (CAL-101) or PI3Kγ (AS252424) inhibitors [13^•^]. The relative contribution of PI3Kδ and PI3Kγ to mast cell function is still controversial: while some studies found PI3Kγ signalling to be critical for mast cell infiltration and degranulation, with transient inhibition of p110γ with NVS-PI-4 sufficient to prevent mast cell extravasation in a passive cutaneous anaphylaxis (PCA) model [14,15], another study showed an essential role for PI3Kδ, but not PI3Kγ, signalling in PCA induced mast cell extravasation [16]. PI3Kβ, PI3Kδ and PI3Kγ also contribute to optimal dendritic cell (DC) and macrophage function [1,17].

PI3K signalling can promote pro-inflammatory cytokine production through NFκB activation downstream of AKT and mediate IL-6 secretion in response to CD80/CD86 stimulation in DC [18]. However, PI3K also play a regulatory role in certain innate immune responses. Several studies identified an inhibitory role for PI3K signalling in TLR mediated inflammation: PI3Kδ activation downstream of TIRAP-MyD88 dependent (TLR2, TLR4) and TRAM-TRIF dependent (TLR4, TLR3) stimulation inhibits pro-inflammatory cytokine secretion while increasing the production of IL-10 in macrophages and DC [19–24]. Possible mechanisms are thought to be through AKT-dependent inhibition of GSK3β, leading to increased levels of CREB and competitive inhibition of NFκB-p65 and AKT dependent inhibition of FoxO1 [23,24]. TLR4 is unique in being activated via a TIRAP dependent mechanism on the cell membrane and also via TRAM following endocytosis. PI3Kδ can mediate a switch between TIRAP dependent pro-inflammatory cytokine secretion and TRAM-dependent IL-10 secretion, thereby limiting inflammation and protecting mice from LPS induced endotoxic shock [21]. PI3Kδ can also control type I IFN production by regulating IRF-7 nuclear translocation in human plasmacytoid DC [25]. PI3Kδ could therefore be a promising therapeutic target in diseases where this pro-inflammatory response is dysregulated. Physiologically, PI3K is regulated by phosphatase and tensin homolog (PTEN) which reverts PIP_3_ to PI(4,5)P_2_. Myeloid cell-specific PTEN deficiency leads to increased PIP_3_ levels, reduced inflammation, increased macrophage phagocytic ability and resistance to infection in mice [26]. Similarly, aged macrophages show increased expression of PI3Kδ with decreased pro-inflammatory cytokine production in response to TLR stimulation, which is partially reversed by the pan-class I PI3K inhibitor LY924002 [20]. Recently it was also shown that LY924002 can reduce TLR3 dependent IL-10 secretion in BCG infected macrophages [22].

**Figure 2:**
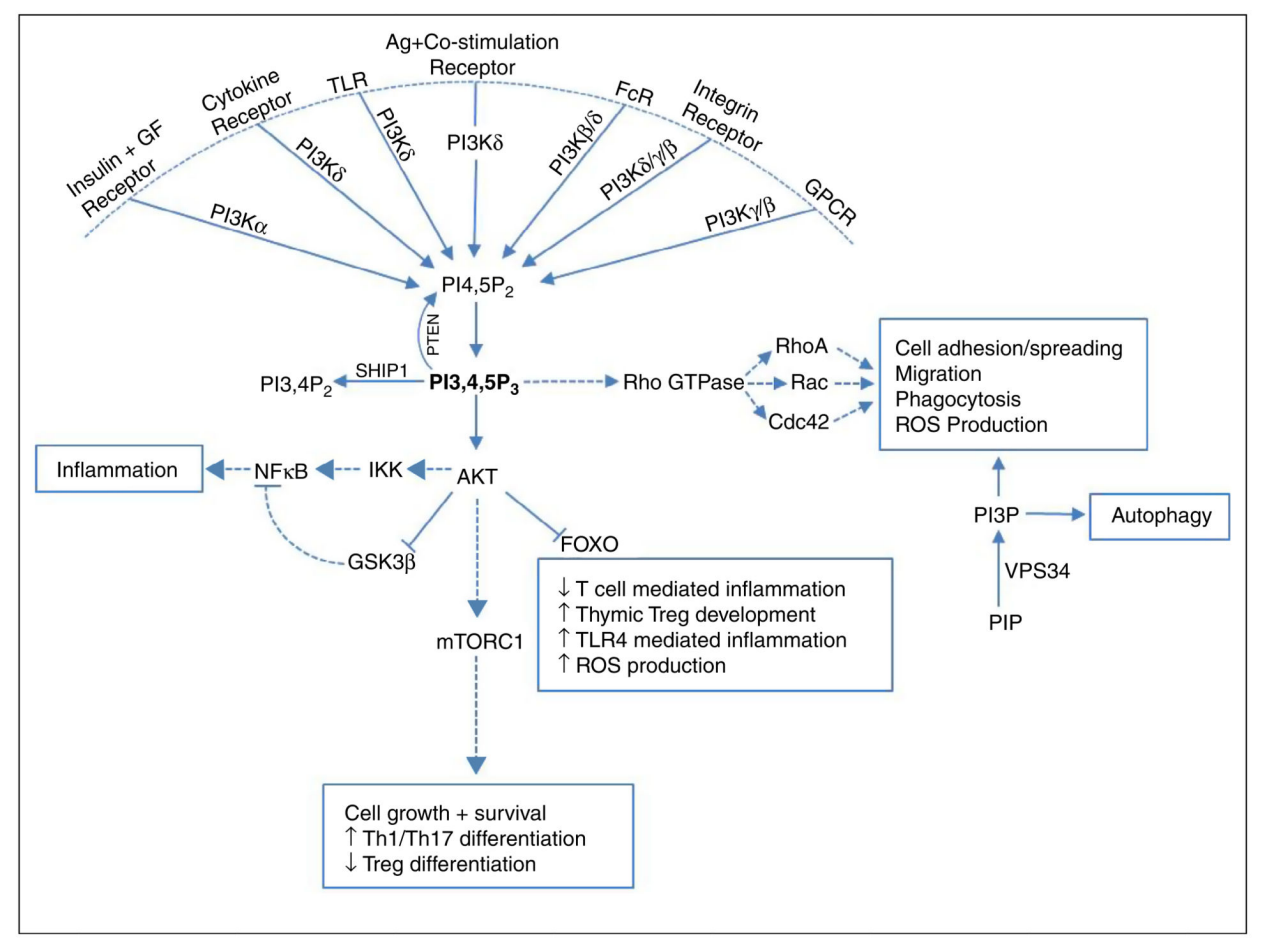
Summary of positive and negative immune regulation by PI3Ks. PI3K signalling play a role in positive and negative regulation of immune cell effector functions, and the outcome of inhibition will depend on inhibitor selectivity and disease context.

Together, these studies show that the PI3K/AKT signalling pathway plays a complex role in orchestrating both pro-inflammatory and anti-inflammatory pathways to maintain effective immunity while protecting host tissues (Figure 2).

## Targeting class I PI3K in autoimmune and inflammatory disorders

Autoimmune disease results from a breakdown in tolerance leading to an immune response directed against host cells, causing conditions such as multiple sclerosis (MS), systemic lupus erythematosus (SLE), rheumatoid arthritis (RA), psoriasis and autoimmune (type I) diabetes. Chronic inflammatory conditions such as chronic obstructive pulmonary disease (COPD), atherosclerosis and inflammatory bowel disease (IBD) arise from failure to resolve an ongoing immune response [1,27]. Several of the driving factors of COPD and atherosclerosis have been identified. In atherosclerosis patients oxidised LDL promotes arterial inflammation, while in a large number of COPD patients cigarette smoke contributes to the pathogenesis. However, it is clear that pathogen-driven responses trigger exacerbations in COPD patients which lead to worsened inflammation and a general decline in health status [28]. Allergic conditions such as asthma or anaphylaxis are caused by an inappropriate immune response directed against a normally harmless antigen [1]. Uncontrolled inflammation is also a risk factor for the development of cancer, and has been shown to contribute to tumour growth and metastasis [5].

PI3Kδ and PI3Kγ are extensively studied as potential targets for anti-inflammatory treatments and the fact that these isoforms have complementary roles in many aspects of immune function provides a clear rationale for the therapeutic use of PI3Kδ and/or PI3Kγ inhibitors (see Table 2 for inhibitors in clinical trials and Table 3 for inhibitors used in pre-clinical models). Indeed, inhibiting PI3Kδ and PI3Kγ in different mouse models of inflammatory disease produced promising results: the dual PI3Kδ/γ inhibitor TG100-115 reduced inflammatory cell infiltrates in an OVA-induced asthma model as well as in smoke-induced and LPS-induced models of airway inflammation when administered as an aerosol [29]. More recently another dual selective PI3Kδ/γ inhibitor, IP-145 (duvelisib), administered systemically also reduced eosinophil infiltration in an OVA-induced asthma model [30^••^]. Interestingly, these preclinical models show that both inhaled and systemic administration routes are effective. Selective PI3Kδ inhibition was found to restore glucocorticoid sensitivity in smoke-induced COPD models by preventing tyrosine nitration of HDAC-2 [31] and IC87114, a selective PI3Kδ inhibitor, reduced inflammatory cell infiltrates and IL-17 secretion in an OVA-induced asthma model [32]. PI3Kδ kinase dead mice are also protected against OVA-induced airway eosinophilia due to decreased Th2, but not Th1 mediated inflammation [33]. Collectively, these data show that class I PI3K signalling may play a key role in the pathogenesis of COPD and asthma [28,34]. This is strengthened by the observation that aberrant migration and decreased accuracy of human neutrophils derived from COPD patients is corrected by PI3Kδ inhibition [35]. However, increased neutrophil survival is also an important aspect of COPD and this was not influenced by isoform-selective PI3K inhibition [36]. GSK recently developed an inhaled p110δ inhibitor GSK2269557 which is currently in phase 2 clinical trials for COPD and asthma (NCT02294734). Another approach to PI3Kδ inhibition is being developed by Aquinox: their SHIP-1 activator AQX-1125 is being tested in a phase 2 study in exacerbating COPD patients (NCT01954628).

Experimental autoimmune encephalitis (EAE) is a model for multiple sclerosis. EAE progression is mainly driven by Th17 mediated inflammation of the CNS leading to the destruction of myelin, with antigen presenting cells (APC) playing a key role in the amplification of inflammation [37]. Genetic and pharmacological inhibition of PI3Kγ significantly reduced CNS inflammation and disease progression [38,39], while PI3Kδ kinase dead mice also showed reduced disease severity in conjunction with a defective Th17 response [40]. However, PI3Kδ signalling is also essential for the optimal development and function of Treg [10,11,41^••^]. In fact, our data indicate that despite reduced Th17 and Th1 responses, p110δ kinase dead mice are not protected against EAE progression, likely due to a concomitant reduction in Treg (A Stark, E Slack, K Okkenhaug, unpublished). Furthermore, PTEN deficient macrophages show increased expression/secretion of arginase I, which could inhibit the pro-inflammatory effects of DC and T cells and protect mice against EAE [42]. Psoriasis is also a Th17 driven disease and may benefit from PI3Kδ and/or PI3Kγ inhibition. Imiquimod-induced skin inflammation was reduced in PI3Kγ deficient and PI3Kδ kinase dead mice, while PI3Kδ (IC87114) and PI3Kγ (AS605240 and AS614006) inhibitors reduced pro-inflammatory cytokine secretion in human CD4^+^ memory T cells and PBMC from psoriasis patients [43]. Inhibiting PI3Kδ using IC87114 improved graft survival in a mouse heart transplant model [44] and delayed disease progression the NOD mouse model of diabetes [45].

PI3Kδ and PI3Kγ inhibition also attenuate disease progression in mouse models of SLE [46–50]. SLE is driven by autoreactive T cells and B cells, with renal immune complex deposition and macrophage driven inflammation key features of the disease. Treatment of MRL/*lpr* mice with the PI3Kδ selective inhibitor GS-9829 reduced kidney damage and prolonged life span. GS-9829 decreased effector-memory T cells and serum IL-6 and TNF-α levels, and also reduced macrophage infiltration in the kidneys [48]. These results were corroborated by another study reporting that the PI3Kδ selective inhibitor MSC2360844 can inhibit pro-inflammatory cytokine secretion by B cells, T cells and DC, and improve renal disease in a NZBW F1 mouse model [49]. Interestingly, haploinsufficient p110δ^WT/D910A^ showed resistance to an autoreactive B cell driven lupus-like syndrome when crossed to a Lyn^−/−^ background, by a mechanism that appear to involve attenuated T cell function [50]. Treatment with the PI3Kδ inhibitor IC87114 also improved disease outcome in the BXSB model of SLE [46] and the PI3Kγ inhibitor AS605240 was effective in reducing disease severity and increasing life-span in MRL/*lpr* mice [47]. Furthermore the dual p110δ/p110γ inhibitor IP-145 inhibited disease progression the NZBWF1/J mouse model of SLE [30^••^].

Inhibitors of PI3Kδ, PI3Kγ and dual selective inhibition are also effective in alleviating the symptoms of RA in animal models. The PI3Kγ inhibitors AS605240, TASP0415914 and CZC24823 reduced the development of collagen induced arthritis (CIA) [39,51,52], and genetic as well as pharmacological inhibition improved symptoms in the effector phase K/BxN serum transfer and αCII models, mainly driven by neutrophilic inflammation [52,53]. Neutrophil migration to LTB_4_ is markedly reduced by dual PI3Kγ/δ inhibition compared to inhibition of either isoform alone [53]. However, while the dual PI3Kγ/δ inhibitor IP-145 could significantly reduce ankle swelling in a rat CIA model [30^••^], it did not improve RA scores in a recent phase 2 clinical trial, showing that animal models do not always predict clinical outcomes in patients. Using the K/BxN mouse model, a separate study show reduced disease development in PI3Kβ deficient mice at low, but not high doses of serum transfer, while additional PI3Kδ deficiency markedly reduced disease severity at high serum transfer doses, indicating a role for dual PI3Kδ/PI3Kβ inhibitors in this context [7].

ZSTK474 is a pan-class I PI3K inhibitor, and was also found to reduce inflammation and disease progression in RA and EAE mouse models [54,55]. However, there is a greater risk of adverse side effects when inhibiting PI3Kα and PI3Kβ in addition to PI3Kδ and/or PI3Kγ. Results from clinical trials show that pan-class I inhibitors are associated with hyperglycaemia, gastrointestinal and psychiatric effects [56]. Moreover, pan-class I inhibitors do not necessarily control inflammation better than dual PI3Kδ/PI3Kγ inhibitors [57].

PI3Kδ and PI3Kγ single and dual isoform selective inhibitors are generally well tolerated in mouse models, and mice deficient in p110δ or p110γ do not show overt clinical phenotypes despite established immunological defects. There is considerable redundancy among the PI3K isoforms and not all immune functions are PI3K dependent. Therefore, selective inhibition is likely to blunt, rather than completely ablate immune function. Mice are normally kept under specific pathogen free (SPF) conditions and are not exposed to common pathogens and co-morbidities; therefore potential increased susceptibility to infection needs to be considered in human trials [58]. Serious side effects were reported for patients treated with the PI3Kδ selective inhibitor idelalisib which included neutropenia, pneumonitis, colitis, diarrhoea and evidence of liver damage as indicated by the black box label attached to Zydelig (Idelalisib) [59^••^,^60^]. Among these, colitis appears to be the most common and it is worth noting that the kinase dead p110δ^D910A^ mice predicted PI3K inhibition can cause colitis [61]. The side effects associated with idelalisib suggest that transient, low dose, or local administration such as inhalation of PI3Kδ inhibitors should be considered to manage inflammatory conditions where possible.

## Increased class I PI3K signalling is a cause of primary immunodeficiency

Recently, autosomal dominant gain of function mutations of PIK3CD (encoding p110δ) and PIK3R1 (encoding p85α) were described in individuals diagnosed with primary immune deficiencies [62^••^,63^••^,^64^–^67^]. These patients suffer from severe recurrent respiratory infections and have increased susceptibility to lymphoma. B cells from the patients were defective in immunoglobulin class switching. Many patients also presented with T cell lymphopenia associated with increased numbers of senescent T cells. Stimulation of patient T cells resulted in low cytokine production and increased activation-induced cell death, which could be partially rescued by the addition of IC87114 which also reduced PIP_3_ levels [62^••^,^66^]. These results indicate that idelalisib, or other PI3Kδ inhibitors under development, could significantly improve the outcome of immune-deficient patients with activating p110δ or p85α mutations. Also, in one patient, rapamycin treatment restored normal T cell populations [63^••^]. It remains to be determined whether an oral or inhaled route of administration would be preferable in these severely affected patients, and this is likely to depend on the disease profile of the individual patient and the specific side effects associated with each route.

## Class I PI3K and cancer

The PI3K/AKT/mTOR pathway is of critical importance in tumour development and PIK3A (encoding p110α) as well as PTEN are among the most frequently mutated in human cancers. This provides a strong rationale for pan-class I as well as PI3Kα and PI3Kβ selective inhibition in treating solid cancers expressing these isoforms. Initially this strategy was met with limited success, mainly due to dose-limiting side effects and development of resistance due to negative feedback mechanisms activating alternative survival pathways. These issues can be addressed by combination-therapies inhibiting several signalling nodes at once, and current strategies for targeted inhibition of PI3Kα and PI3Kβ were recently reviewed [5,56,68]. PI3Kδ and PI3Kγ are potential targets in haematological cancers, and a notable success is the development of idelalisib which has shown remarkable efficacy in treating Chronic Lymphocytic Leukaemia (CLL) and non-Hodgkin’s lymphoma, and is now approved for clinical use [59^••^,^60^,69^•^].

In addition to targeting the PI3K pathway to inhibit tumour cell growth directly, PI3K inhibitors may also be used to improve anti-tumour immune responses. Genetic or pharmacological inhibition of PI3Kδ (PI-3065) reduced tumour burden and metastasis in a range of mouse cancer models including melanoma, thymoma, lung, breast and pancreatic cancer [41^••^]. In these models, PI3Kδ inhibition attenuated Treg function and tumour infiltration while leaving the cytotoxic T cell response relatively unscathed, resulting in enhanced anti-tumour immunity. PI3Kδ inhibition can also alleviate graft versus host disease while maintaining strong graft versus leukaemia effect [70].

Genetic or pharmacological inactivation of p110γ using TG100-115 and AS605240 was also found to reduce tumour growth and metastasis in melanoma, lung, pancreatic and breast cancer models. PI3Kγ signalling was required for myeloid cell recruitment to the tumour microenvironment through integrin α4β1 mediated adhesion, in response to growth factors and chemokines. Therefore, inhibition of p110γ signalling was effective in reducing general tumour associated inflammation and angiogenesis without affecting systemic numbers of myeloid cells [4].

Dual p110δ/p110γ inhibitors are already in clinical trials for haematological cancers, and are effective in controlling inflammation [30^••^]. It would therefore be interesting to evaluate the effect of these compounds on anti-tumour immune responses in solid cancer models.

## Class II PI3K

Class II PI3K phosphorylate PIP and PI4P to form PI3P and PI(3,4)P_2_ respectively. Although the biology of class II PI3K signalling is still incompletely understood recent progress have indicated a role for PI3KC2 isoforms in immune cell signalling and tumour development [71]. However, because selective inhibitors against the class II PI3Ks have yet to be described, we do not consider this class further in this review.

## Class III PI3K

Vps34 phosphorylate PIP to form PI3P at the pre-autophagosome or endosome leading to the recruitment of FYVE and PX domain containing proteins [1,2,72]. Vps34 associates with the protein kinase Vps15 in different protein complexes, and play an important role in membrane trafficking and protein sorting pathways. PI3P produced by Vps34 is critical for autophagosome and phagosome maturation as well as NOX2 mediated ROS production, thereby playing a key role in autophagy, as well as pathogen uptake and killing by innate immune cells.

Autophagy maintains normal cell function by removing misfolded proteins and damaged organelles, but also has specialised functions in the immune system. Autophagy mediates intracellular TLR activation by bringing cytoplasmic antigens in contact with TLR in the lysosome, and promotes cross-presentation of intracellular antigens on MHCII [2]. T cell-specific loss of Vps34 impairs invariant NKT cell development and peripheral T cell homeostasis, which ultimately lead to intestinal inflammation and wasting syndrome as a result of Treg dysfunction [73].

Recently three independent groups published selective Vps34 inhibitors: SAR405, PIK-III and Vps34-IN1 [74^•^,75^•^,76^•^,^77^]. These compounds will increase our understanding of the functions performed by Vps34 and opens up the possibility to target this kinase for therapeutic benefit. Already, SAR405 was found to act synergistically with the mTOR inhibitor everolimus to reduce proliferation in a renal tumour cell line [74^•^], while PIK-III was used to identify a novel autophagy substrate: NCOA4 binds ferritin and plays a role in recycling iron from red blood cells in the spleen [75^•^]. VPS34-IN1 revealed that class I and class II PI3K activity contribute to PIP_3_ mediated activation of SGK3 [76^•^]. This opens up the possibility that synergistic class I PI3K and Vps34 inhibitors could be used in the treatment of tumours with elevated SGK3 activity. Whether Vps34 inhibitors have potential for use in immune-mediated diseases remains to be explored: germ-line loss of Vps34 is embryonically lethal, and tissue specific deletion found a critical role for Vps34 in normal neuron, heart and liver function [78]. However, further study is required to establish if systemic Vps34 inhibition will be tolerated.

## Conclusion

Although much progress has been made in understanding the role of PI3K signalling in inflammation and cancer, many questions still remain. PI3K signalling plays a complex and often opposing role in the regulation of immune responses and the effect of inhibiting PI3K is dependent on the context of activation. The factors modulating opposing functions of PI3K signalling are not yet clearly understood and warrant further investigation. This complexity of PI3K pathway regulation poses an interesting challenge for the therapeutic application of PI3K inhibitors: a better understanding of which isoforms are critical in different disease mechanisms and to what extent inhibition is favourable or not is essential. Animal models and early clinical trials show great potential in therapeutic targeting of this pathway in immune-related disorders and cancer, but do not always predict clinical efficacy.

## Figures and Tables

**Table 1:** Summary of PI3K classes

	Isoforms	Tissue distribution	Substrate	Product	Adaptor molecules
Class IA	p110α	Ubiquitous	PI(4,5)P_2_	PI(3,4,5)P_3_ aka PIP_3_	p85α(p50α,p55α) p85β; p55γ
	p110β	Ubiquitous			
	p110†	Leukocytes, neurons			
Class IB	p110γ	Leukocytes, cardiac myocytes	PI(4,5)P_2_	PI(3,4,5)P_3_	p101/p84/p87
Class II	C2α	Epithelium, endothelium	PI, PI4P	PI3P, PI(3,4)P_2_	
	C2β	Ubiquitous			
	C2γ	Hepatocytes			
Class III	VPS34	Ubiquitous	PI	PI3P	VPS15(p150)

**Table 2:** Class I PI3K/mTOR inhibitors in clinical trials

Compound	Target	Indication	Clinical trial identifier
BYL-719	p110α	Recurrent or Metastatic Squamous Cell Carcinoma	NCT02145312, Phase 1/2
MLN1117 (INK-1117)	p110α	Advanced Nonhaematologic Malignancies	NCT01899053, Phase 1b
AZD6482	p110β	Antiplatelet Effect	NCT00853450, Phase 1
AMG 319	p110δ	Haematologic Malignancies	NCT01300026, Phase 1
GSK2269557	p110δ	COPD	NCT02294734, Phase 2
Idelalisib (CAL-101)	p110δ	Chronic Lymphocytic Leukaemia (CLL) Non-Hodgkin Lymphoma	FDA and EMA approved, 2014
INCB040093	p110δ	B cell malignancies	NCT01905813, Phase 1
TGR-1202	p110δ	Cancer (CLL and B-cell lymphoma)	NCT01767766, Phase 1
UCB-5857	p110δ	Psoriasis	NCT02303509, Phase 1
AZD8835	p110α/p110δ	Advanced Solid Malignancies	NCT02260661, Phase 1
BAY80-6946 (Copanlisib)	p110α/p110δ	Non-Hodgkin’s Lymphoma	NCT01660451, Phase 2
GDC-0941 (Pictilisib)	p110α/p110δ	Breast Cancer	NCT01437566, Phase 2
AZD8186	p110β/p110δ	Prostate, Lung and Breast Cancer	NCT01884285, Phase 1
GS-9820 (Acalisib)	p110β/p110δ	Lymphoid Malignancies	NCT01705847 Phase 1b
IPI-145 (Duvelisib)	p110δ/p110γ	Non-Hodgkin Lymphoma Small lymphocytic lymphoma; CLL	NCT01882803, Phase 2 NCT02004522, Phase 3
RP-6530	p110δ/p110γ	Haematologic malignancies	NCT02017613, Phase 1
RV-1729	p110δ/p110γ	Asthma/COPD	NCT01813084, Phase 1
BKM120	pan-class I	Metastatic Breast Cancer	NCT01633060, Phase 3
XL-147 (SAR245408)	pan-class I	Malignant neoplasm	NCT01587040, Phase 1
ZSTK474	pan-class I	Advanced Solid Malignancies	NCT01682473, Phase 1
BEZ235	pan-class I/mTOR	Renal Cancer	NCT01453595, Phase 1/2
BGT226	pan-class I/mTOR	Solid Tumours, Breast Cancer	NCT00600275, Phase 1/2
GSK2126458	pan-class I/mTOR	Solid Tumours Pulmonary Fibrosis	NCT00972686, Phase 1 NCT01725139, Phase 1
VS-5584	pan-class I/mTOR	Non-haematologic metastatic cancer Lymphoma	NCT01991938, Phase 1
XL-765 (SAR245409)	pan-class I/mTOR (p110γ)	Malignant neoplasm	NCT01587040, Phase 2
PX866	pan PI3K	Metastatic prostate cancer	NCT01331083, Phase 2
SF1126	pan PI3K	Neuroblastoma	NCT02337309, Phase 1
AQX-1125	SHIP1 activator	COPD Atopic Dermatitis Interstitial Cystitis	NCT01954628, Phase 2 NCT02324972, Phase 2 NCT01882543, Phase 2

**Table 3:** Some isoform-selective PI3K inhibitors used in pre-clinical studies: IC50 μM

Compound	Target	p110α	p110β	p110δ	p110γ	Vps34	Ref.
A66	p110α	0.032	0.236	1.25	3.48		[1]
NVS-PI3-2	p110α	0.075	5.5	0.98	2.4		[36]
PW12	p110α	0.015	0.83	0.73	0.97		[17]
HBC-417	p110β	0.38	0.007	0.03	0.2		[36]
TGX-115	p110β	61	0.13	0.63	>100		[17]
TGX-221	p110β	5	0.007	0.1	3.5		[1]
AS252424	p110γ	1.07	>20	>20	0.035		[12]
AS614006	p110γ	1.68	0.062	0.166	0.003		[43]
AS605240	p110γ	0.06	0.27	0.3	0.008		[1] [52]
CZC24832	p110γ	>10	1.1	8.2	0.027		[80]
NVS-PI3-4	p110γ	1.8	0.25	0.75	0.09		[36]
TASP0415914^a^	p110γ				0.029		[51]
GS-9820	p110δ	5.441	3.377	0.012	1.389	12.685	[79]
GS-9829	p110δ	>10	>10	0.0703	>10	>10	[48]
IC87114	p110δ	>100	±5	0.1	±1		[1]
NVS-PI3-3	p110δ	0.18	0.6	0.003	0.31		[36]
PI-3065	p110δ	0.91	0.6	0.005	>1000		[41^••^]
YM-024	p110α/p110δ	0.3	2.65	0.33	9.07		[36]
TG100-115	p110δ/p110γ	1300	1200	0.235	0.083		[81]
PI-103	pan-class I	0.0008	0.088	0.048	0.15		[36]
wortmannin	pan-PI3K/mTOR	0.001	0.01	0.005	0.009		[36]
LY294002	pan-PI3K/mTOR	0.7	0.306	1.33	7.26		[36]
PIK-III	VPS34	3.96	>9.1	1.2	3.04	0.018	[75^•^]
SAR405	VPS34	>10	>10	>10	>10	0.0012	[74^•^]
VPS34-IN1	VPS34	8.036	21.44	1.896	2	0.025	[76^•^]

a No selectivity data published.
